# THE NEED FOR FAMILY-CENTERED HOME CARE IN HOME-BASED DEMENTIA CARE

**DOI:** 10.1093/geroni/igad104.1524

**Published:** 2023-12-21

**Authors:** Jennifer Reckrey, Deborah Watman, Emma Tsui

**Affiliations:** Icahn School of Medicine at Mount Sinai, New York City, New York, United States; Icahn School of Medicine at Mount Sinai, New York City, New York, United States; CUNY Graduate School of Public Health, New York, New York, United States

## Abstract

Family caregivers, paid caregivers, and geriatricians frequently work together to care for people with dementia living at home, yet little is known about the paid caregiver role in home-based dementia care. We conducted repeated, one-on-one, semi-structured interviews with the care team (i.e., family caregiver, paid caregiver, geriatrician) of 9 people living at home with dementia over 6 months to explore multiple perspectives on paid caregivers’ role in care over time. In all, 29 unique respondents participated in 75 interviews that were analyzed using the framework method of analysis. Results revealed nuanced and highly individual care arrangements where paid caregiver roles in care varied significantly, though changes to this role over time were minimal. Families were the key players in defining the paid caregivers’ role regardless of home care agency involvement or source of paid caregiver payment (i.e., Medicaid-funded vs. privately paid) and geriatricians frequently deferred to family caregivers to determine how they should engage with paid caregivers. While paid caregivers often described their emotional connection to their clients, family caregivers and geriatricians rarely described this as an important aspect of paid care. To meet the nuanced care needs of people with dementia, home care needs to be centered around the unique needs of both people with dementia and their family caregivers. Rather than provide prescriptive standards for home care, family-centered home care should facilitate improved communication and clear expectation setting to be sure that care best meets the unique care needs of people with dementia living at home.

